# Gender roles in maize food systems: a case of smallholder farmers in rural Malawi

**DOI:** 10.3389/fsoc.2026.1938286

**Published:** 2026-10-21

**Authors:** Victor Munkhuwa, Chimwemwe Chilenga, Yananda Mandhlopa, Tinna Ng’ong’ola-Manani, Agnes Mbachi Mwangwela, Kingsley George Masamba

**Affiliations:** 1 Department of Food Science and Technology, Lilongwe University of Agriculture and Natural Resources, Lilongwe, Malawi; 2 Department of Human Ecology, Lilongwe University of Agriculture and Natural Resources, Lilongwe, Malawi

**Keywords:** food security, food systems, gender, maize, smallholder farmers

## Abstract

The purpose of this study was to determine gender roles of smallholder farmers in maize food systems of rural Malawi. Gender roles and perception levels in the maize food system were assessed using a convergent mixed methods research design whereby both qualitative and quantitative approaches were used. Results showed that the majority of members in farmer groups and cooperatives (85%), loan beneficiaries (59%), and farm inputs beneficiaries (64%) are men. This means that males are the dominant gender in the farmer groups and cooperatives and that they have an upper hand in terms of accessing resources that are critical for maize production when compared to women. Men take a major role in deciding on how to access farm inputs (54%) and how to use the farm inputs (50%). Men and women jointly take major roles in deciding on the type of seed to grow (40%) and on the use of loan money (35%). This shows that men take the lead in decision-making processes of their households. Furthermore, women are less involved than men when it comes to implementing activities during the maize production, processing, and distribution stages. However, there is higher involvement of women than men when it comes to activities during the maize consumption stage. The correlation test has shown weak linkages and little influence between the maize food system stages. Stakeholders should promote adequate gender integration in all the maize food system stages to ensure equal participation and benefits for men and women and improve food security.

## Introduction

Attainment of food security in populations depends on several factors that revolve around food system stages of food production, processing, distribution and consumption by households (Amao et al., 2023; FAO et al., 2019; Maziya et al., 2017; Tesfaye et al., 2023). Gender integration during food system stages is one vital factor that enables men and women to equally have access to resources, participate in food value chains, marketing and utilization of their produce (Jomo, 2026; Kini, 2022; Mawia et al., 2025). However, despite both men and women being engaged in agricultural activities, women still face various hindrances to their full involvement in the food system stages (Kihiu and Amuakwa-Mensah, 2021). Such limitations include unresponsive policies and structures to the varying needs of men and women; inadequate start-up and maintenance resources to support enterprises; inadequate mobility and security; negative stereotyping of women in perceived male dominated fields; inadequate information and training; poor technological skills and knowledge; and, inadequate time (Benjamin and Meyers, 2016; Forsythe et al., 2016; Hokoza, 2025; Said-allsopp and Tallontire, 2015).

In many value chains and places women face gender-related hindrances to their full involvement in food system stages due to social, cultural, economic, and gender-based inequitable conditions that prevent them from participating fully (Begho et al., 2025; Djurfeldt et al., 2018; Forsythe et al., 2016). A related study (Quisumbing et al., 2021) reported that women from Malawi, Benin, the Philippines, and Bangladesh had low level participation in agricultural value chains due to sociocultural norms and other context factors. Other similar studies (Kehinde and Kehinde, 2020; Radchenko and Corral, 2018) have argued that equal involvement of men and women along the food system stages would assist in ensuring equal participation and benefits in the development of sustainable food systems and food security. The authors observed that market participation amongst farmers can enhance agricultural productivity, increase their financial income and food security, and lower their poverty levels. Furthermore, another study (Das et al., 2023) has also linked increased women’s involvement in food system stages to improved dietary diversity and nutrition outcomes for households.

In Malawi, maize is the main staple crop grown and consumed by the majority of households. Ensuring adequate gender integration in the maize food system is critical in promoting food security within the Malawian communities. It is reported that smallholder farmers, especially women, remain disadvantaged in the value chains as they are unable to process their products (Adam et al., 2020; Donkor et al., 2022). It was therefore necessary for this study to assess gender roles of smallholder maize farmers and perception levels of gender integration in rural Malawi. Findings from this study will help stakeholders in the agriculture sector in advocating for women’s inclusion in the maize food system. Adequate gender integration is critical in ensuring equal participation and benefits for men and women and increased food security in the communities. The findings will also benefit extension workers and academicians, as they will provide additional literature to the already available information on gender roles in the maize food system. The study was conducted in five selected districts of Malawi, which were all the Sustainable Food Systems in Malawi (FoodMa) project’s implementation impact areas. The FoodMa project aimed to strengthen local food systems and improve food security, income, and climate resilience for rural households in the six Malawian districts by 2025.

## Materials and methods

### Study area and study population

The study was implemented in Mhuju Extension Planning Area (EPA) in Rumphi district, Bwengu EPA in Mzimba north district, and Manyamula EPA in Mzimba south district from the northern region of Malawi, and, Santhe EPA in Kasungu district, Chivara EPA in Dowa district, and, Chiosya EPA in Mchinji district from the central region of Malawi (Figure 1). In the targeted areas, the study engaged household heads of smallholder farmers in the rural areas that had been growing maize for at least 3 years.

**Figure 1:**
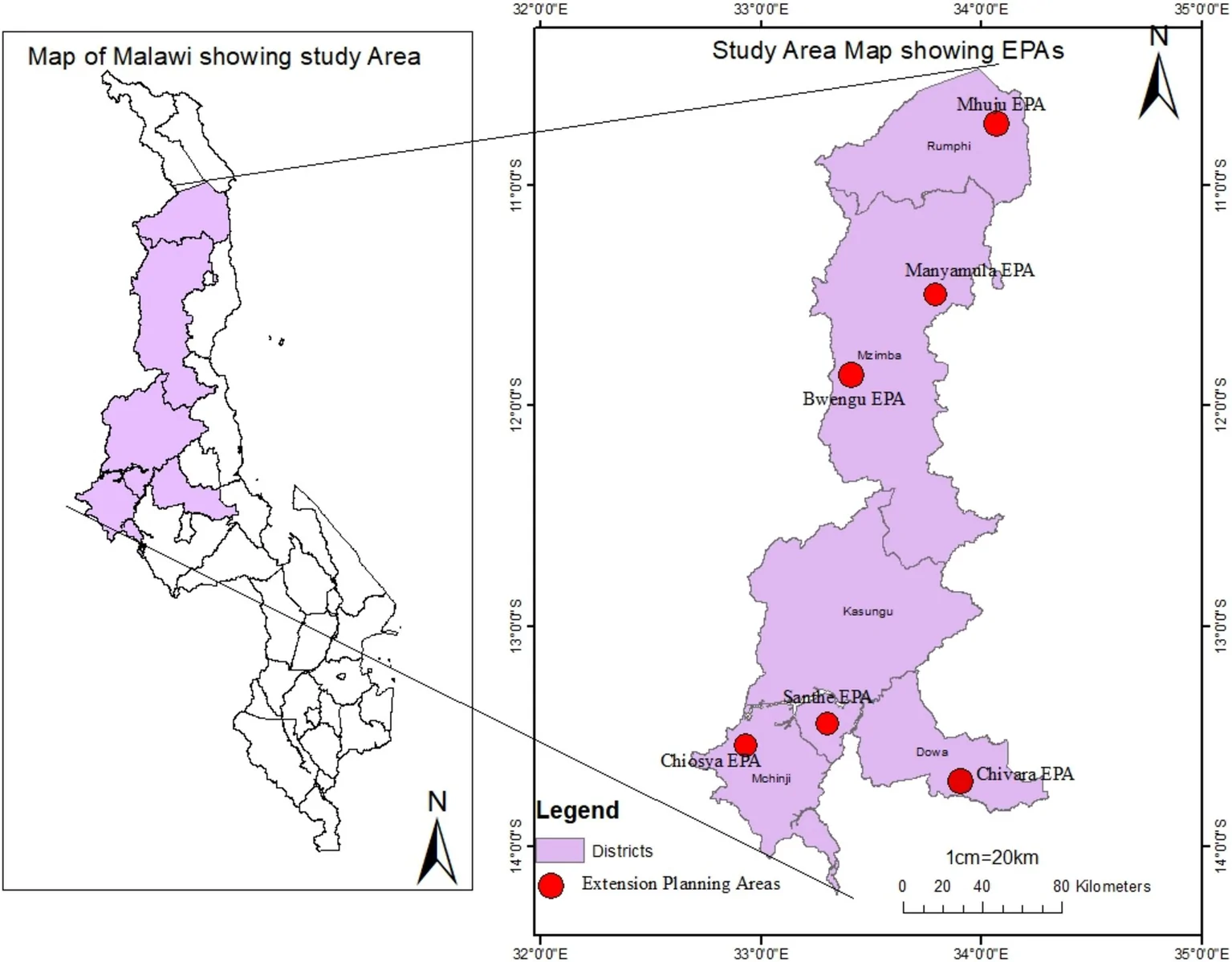
Map of Malawi showing study areas.

### Research design

This study used a convergent mixed methods research design (one-phase design) whereby both qualitative and quantitative approaches were used. A mixed methods research design best suits this study as it addressed the research problem from both narrative and numeric aspects simultaneously (Fetters et al., 2013). This was also appropriate and efficient in ensuring both depth and breadth in data collection in the face of limited time and resources. The quantitative component measured variables such as gender composition, access to resources, participation levels, and household decision-making patterns, while the qualitative component explored experiences, perceptions, social norms, and contextual explanations underlying these quantitative patterns. Figure 2 is an illustration of a convergent mixed methods design (one-phase design) adopted by the study.

**Figure 2:**
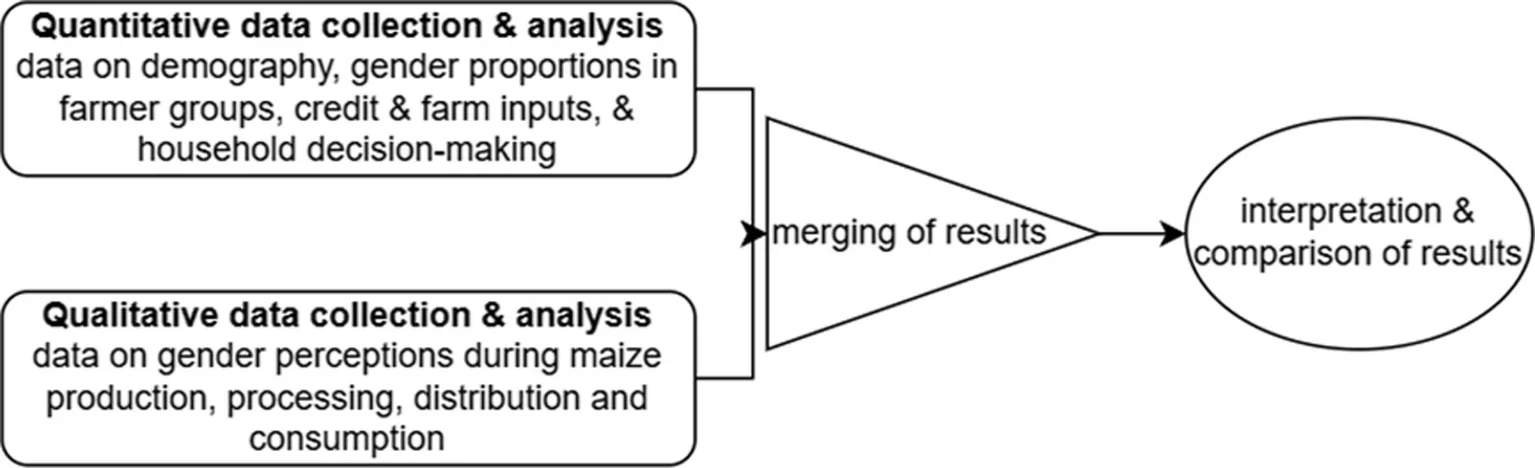
Convergent mixed methods research design (one phase design).

### Sampling technique of study participants

This study used a multistage sampling technique during the identification of respondents to the survey. Firstly, purposive sampling was conducted to identify target districts and EPAs from where the participants were to be drawn. The study purposively selected districts and EPAs that were the impact areas of the FOODMA project in the country. Secondly, random sampling was done to identify the target villages for the interviews. Later, a purposive and convenience sampling technique was used in identifying smallholder maize-farming households. At household level, the study interviewed the heads of the households. The purposive sampling technique is appropriate as it deliberately selects specific individuals that can provide key information with respect to the subject matter. Likewise, convenience sampling is also appropriate as it directs the study to individuals that are readily available and willing to take part in the study at the time of the study visit (Ayhan, 2011). However, convenience sampling may introduce bias, as it depends on easily accessible, non-random participants rather than a representative subset, causing selection bias and undercoverage.

### Determination of sample size

A sample size of 243 households was determined using Yamane’s formula (Yamane, 1967) for finite populations, where *e* = margin of error, *N* = total population, and *n* = sample size. The following assumptions were made: 6% margin of error, 95% confidence level, and an estimated total population of 1,941 households across the 6 selected EPAs. Approximately 40–41 respondents were selected from each of the 6 EPAs using equal allocation. The following formula was used in calculating the sample size.

$$n=\frac{N}{1+N(e)2}$$

### Data collection and analysis

Quantitative data was collected using a designated questionnaire that was uploaded in Kobo Collect software Version 2024.1.3 installed in smartphones through face-to-face interviews with heads of smallholder farming households. Age, sex, name of districts and EPAs, and other demographic characteristics of the participants were also recorded. The collected data was exported to SPSS version 20 for analysis. Categorical data was presented as percentages and the total sample size was reported in all figure captions to clarify the precision of the estimates. Qualitative data was collected through focus group discussions (FGD) with men’s groups and women’s groups using a designated FGD guide. In each EPA, two FGDs were conducted, one with men’s group and the other with women’s group. The data collected was thematically coded to identify themes around the research aim. Key themes emerged, revealing gender integration insights during maize production, processing, distribution, and consumption. The narratives helped to triangulate results that were found from the quantitative aspect of the study. The Pearson correlation coefficient was used to measure an association between the maize food system stages of production, processing, distribution, and consumption variables.

## Results and discussion

### Demographic information of smallholder farming households

According to Table 1, the majority (60%) of the farmers were males and were in the age categories of 21 to 40 years (44%) and 41 to 60 years (38%). The majority of the households’ had 4 to 6 members (60%) and were married (75%). Educational levels were low with 71% attaining primary education, 18% attaining secondary school education, and 1% attaining tertiary education.

**Table 1:** Demographic information of smallholder farming households (*n* = 243).

Variables	Value	Frequency	Percentage
Gender of household head	Male	146	60
	Female	97	40
Age of household head	≤20	7	3
	21–40	106	44
	41–60	93	38
	≥61	37	15
Household size	1–3	58	24
	4–6	145	60
	7–9	40	16
Marital status	Married	183	75
	Widowed	31	13
	Separated	12	5
	Single	9	4
	Divorced	8	3
Education level	Primary	172	71
	Secondary	44	18
	None	24	10
	Tertiary	3	1
Source of livelihood^1^	Farming	238	98
	Casual labour	61	25
	Business	34	14
	Remittances	22	9
	Employment	6	2
Ownership of farming land	Own land	150	62
	Family land	49	20
	Rented land	44	18
Farming land size (hectare(s))	≤ 1	142	58
	>1–≤2	84	35
	>2	17	7
Who manages the farm	Joint man and woman	115	47
	Man	70	29
	Woman	59	24

1 Sources of livelihoods: multiple responses were allowed.

The majority of farmers relied on farming (98%) as their main source of livelihoods, supplemented by casual labour (25%) and businesses (14%). The majority of the households farmed their own land (62%) and operated ≤1 ha farmland (58%). Farm mangement was predominantly done by joint men and women (47%), followed by men only (29%). A related study (Mataka et al., 2023) reported similar trends in terms of particpants age categories, household sizes, marital status, sex of household heads, occupations, and education background.

### Gender composition of farmer groups and cooperatives

Only 40.3% of the farmers indicated that they were aware of the presence of farmer groups and cooperatives in their areas. Out of this, only 20.4% indicated to be members of the farmer groups and cooperatives as of the time of the study. The low levels of awareness and membership can be linked to the inadequate numbers of extension workers available and present in the communities to facilitate the establishment of farmer groups and cooperatives. An analysis of the gender composition of farmer groups and cooperatives data shows that the majority (85%) of the members are males as compared to females (15%) (Figure 3). The FGDs showed that the socioeconomic and cultural factors of the areas favour men in resource ownership and agricultural decision-making; as such, more men are involved in community meetings than women. A similar study (Ingutia and Sumelius, 2022) done in Kenya also reported a small number of female farmers’ members in co-operatives. The authors noted that this deprived most women of access to vital resources such as credit, family planning information, market information, extension visits, and improved water sources, all of which are critical in improving their food security situation.

**Figure 3:**
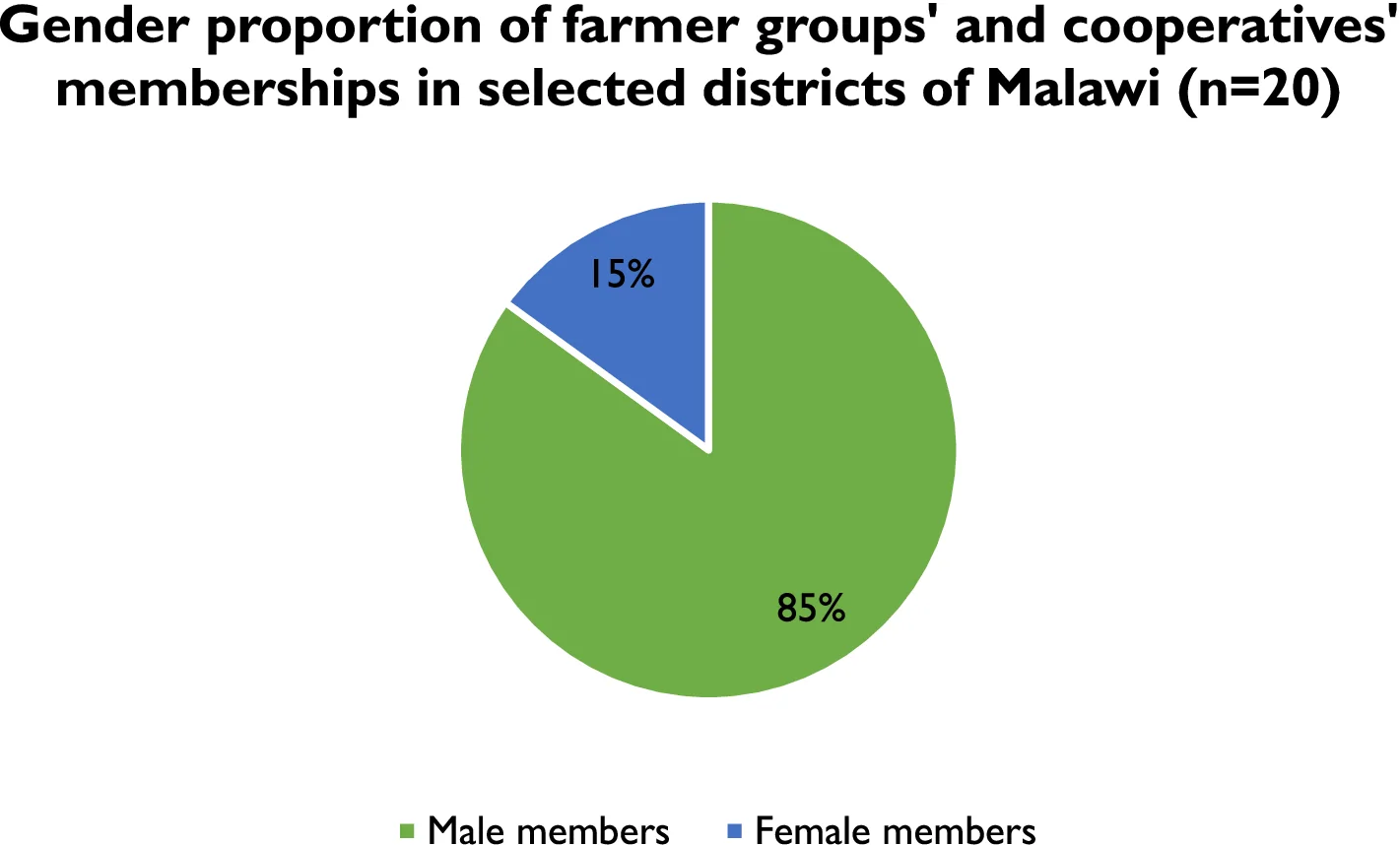
Gender composition of farmer groups and cooperatives.

On average, all farmers had maintained their membership in farmers’ groups and cooperatives for at least 3 years as of the time of the study. The findings show that farmers benefited from the farmer groups and cooperatives in the following ways: having access to joint markets (85%); gaining expertise through extension services (80%); and having access to farm inputs (65%), farm land (10%), and financial loans (10%). Improving women’s participation in farmer groups and cooperatives has a high likelihood of improving their socio-economic life and household food security. Related studies (Bryan et al., 2023; Essilfie et al., 2024; Haque et al., 2024) have also argued that investing in women can improve their household food security and aid in the speedy attainment of sustainable development goals (SDGs).

### Access to financial loans and farm inputs by smallholder farmers

Only 7% of the farmers indicated they had accessed farming loans within the past 3 years as of the time of the study. The low proportion of beneficiaries to farming loans can be attributed to inadequate farming loans-lending institutions and collaterals in the country. In terms of gender composition of the loan beneficiaries, results show that the majority of the loan beneficiaries were men (59%) compared to their counterparts, women (41%) (Figure 4). In FGDs, women said “most women have less access to farm inputs than men do due to their comparative lower financial muscle for accessing the inputs”. Similar studies (Alao et al., 2020; Kehinde and Kehinde, 2020) done in Nigeria also reported higher access rates of farming loans for men than women, as they were endowed with many resources that could be used as collateral. In our study, farmers indicated having accessed their loans from government entities (52%), commercial banks (18%), savings and loans associations (18%), money lenders (6%), and other sources (relatives and friends) (6%) within their communities. Furthermore, farmers indicated they had spent part of their money on farm activities (100%), on their children (24%), on food (24%), and on debts (6%). This expenditure pattern may denote vulnerability as critical needs of farming, children, and foods are all competing for the same shallow pool of funds. Previous studies (Carranza and Niles, 2019; Salima et al., 2023) have also indicated similar patterns of expenditures for smallholder farmers’ credit loans.

**Figure 4:**
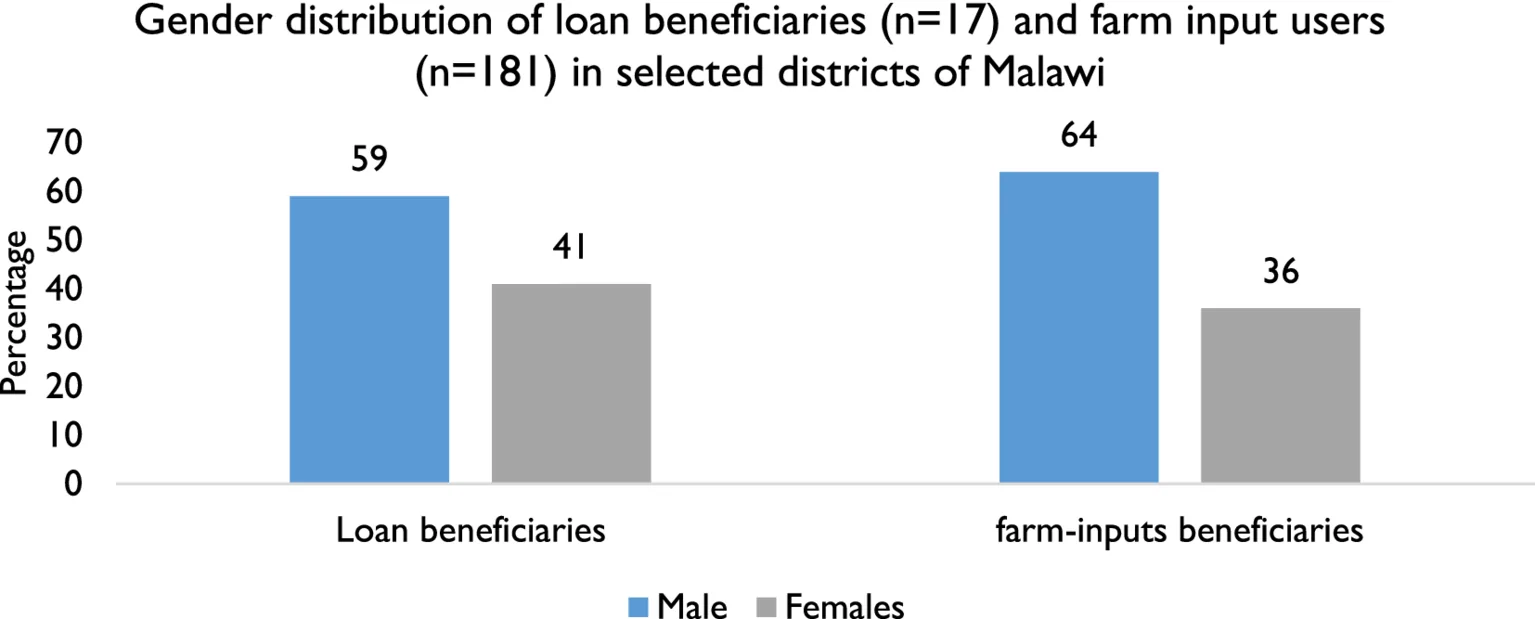
Gender distribution of loan beneficiaries and input users.

Furthermore, the majority (74%) of farmers indicated to have accessed farm inputs within the past 3 years as of the time of the study. A gender analysis of the farm inputs users showed that the majority (64%) of the users were males compared to their counterpart females (36%). Previous studies (Makate and Mutenje, 2021; Mgalamadzi et al., 2024; Ngoma et al., 2021) done in Malawi and Zambia have reported higher intensity usage of improved seed, inorganic fertilizer, improved maize, and joint use of fertilizer and improved seed in male managed plots than in female managed plots. On the types of farm inputs accessed, the beneficiaries indicated having accessed fertilisers (99%), mechanicals (34%), herbicides (8%), pesticides (6%), and insecticides (3%). Sources of these inputs were predominantly government entities (55%), non-governmental organizations (11%), own source (15%), seed companies (10%), and relatives and friends (9%). The results suggest that increasing the number of actors in the farm inputs industry can enhance competition thereby increasing the supply and affordability of inputs for both male and female farmers, thus improving food security.

### Gender levels in decision making of smallholder farming households

Results show that farm input access-related decisions were solely made by men (54%), by women (25%), jointly by men and women (16%), and relatives and friends (4%). The findings also show that farm input usage-related decisions are made solely by men (50%), jointly by men and women (25%), by women (22%), and by relatives and friends (2%) in their households (Table 2). This means that men are more influential than women when it comes to deciding on where to access farm inputs and how to use them at the household level. The FGDs corroborated that ‘men are more financial stable and connected and are as such more able to access farm inputs than women are’. Previous studies (Ngoma et al., 2021; Otieno et al., 2021) have also shown that men possess more wealth and are more networked than women. Higher affluence levels of men than women give them more decision-making power when accessing and utilising farm inputs in their households.

**Table 2:** Gender contributions in decision making of smallholder farming households (%).

Type of decision	Joint men and women	Men	Women	Relatives and friends
Access to farm inputs (*n* = 181)	16	54	25	4
Use of farm inputs (*n* = 181)	25	50	22	2
Type of seed to grow (*n* = 181)	40	35	24	2
Use of loan money (*n* = 17)	35	35	24	1

However, in terms of determining the type of seeds to grow, farmers indicated that jointly men and women (40%), men (35%), women (24%), and relatives and friends (2%) solely make their households’ decisions. The findings further show that men and women jointly (35%), men (35%), women (24%), and relatives and friends (1%), solely make their household decisions on how to spend their loan money. This means that most men and women make joint decisions when it comes to choosing the type of seeds to grow and how to use their loan money. This is a plus in the fight for gender integration, as it gives room for collaborative decision-making. A similar study (Mohammed et al., 2023) done in Ghana observed that joint household decision making arrangements had positive effects on household food security outcomes compared to sole patriarchal decision making arrangements in smallholder farming communities. This shows that encouraging joint decision-making is critical in promoting food systems’ gender integration and attainment of household food security.

### Gender integration perception levels during maize production

Results show that the majority of farmers reported less involvement of women than men when it comes to access to resources (e.g., land, credit and technology) (71%), value and recognition of contributed work (67%), control over proceeds realised from maize production (66%), household decision-making processes throughout the maize production chain (62%), consideration of gender-specific needs and constraints in maize production planning and implementation (57%), access to training, extension services, and market information (56%), and opportunities to participate in farmers' organisations and cooperatives (55%) (Figure 5). This means that men take major roles when it comes to maize production. This was corroborated by the FGDs where women said: “Men have a stronger say on the type of varieties to plant, when to plant the crops and how much land to use than women are”. Inadequate gender integration in the maize production stage has the capacity of derailing maize production productivity due to a lack of coordinated efforts. Previous studies (Badstue et al., 2020; Masuku et al., 2023) observed that rural women were segregated from having access to and owning land as the customary laws favoured men. The authors reported that women were thus excluded from decision-making processes related to productive resources and assets, a situation that contributed to food insecurity. Productive resources are necessary for the sustenance of livelihoods and food security in rural areas (Galiè et al., 2015). In Malawi, there is a need to review the customary laws and fashion them to ensure equal opportunities and benefits for men and women in access to productive resources.

**Figure 5:**
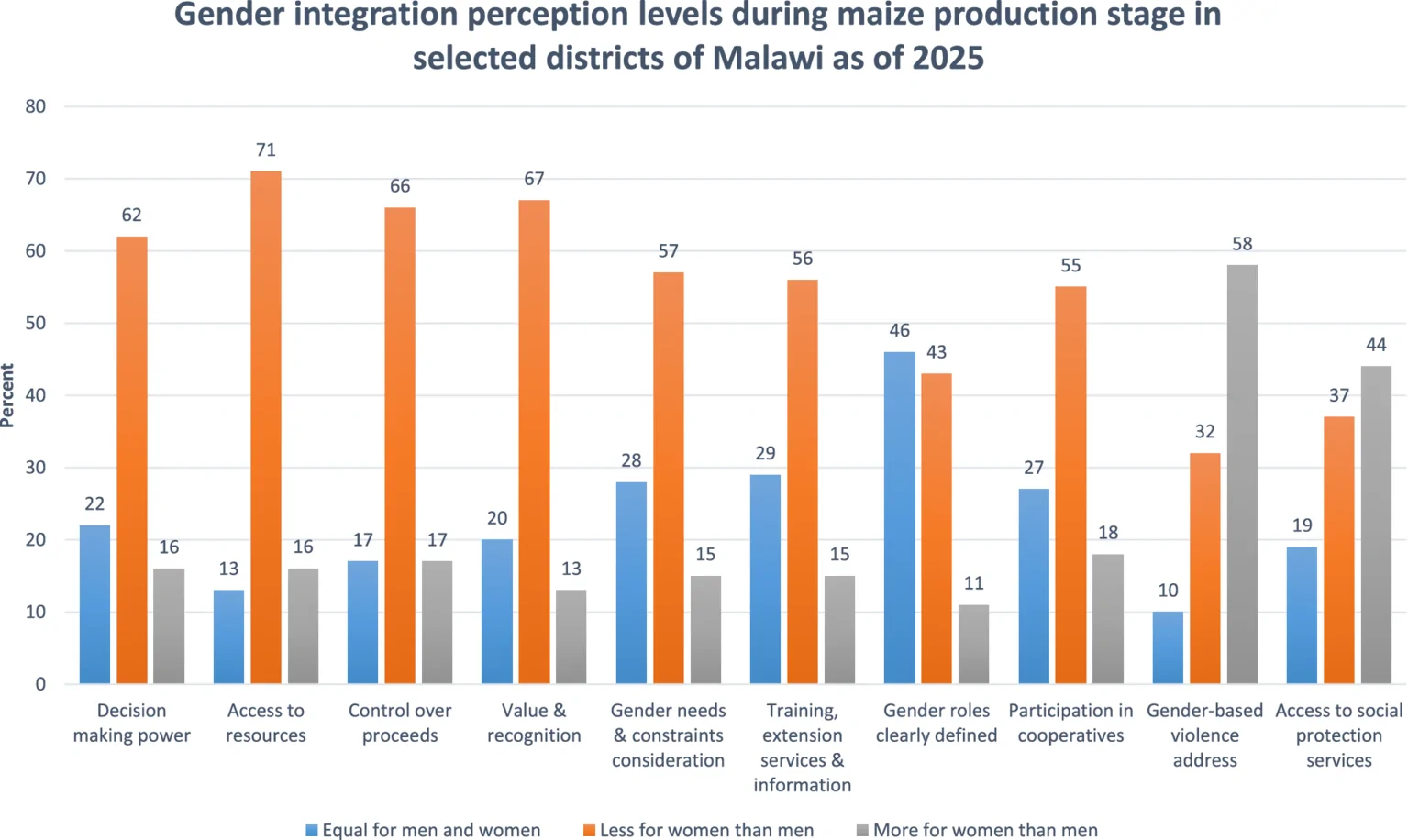
Gender integration perception levels during maize production (*n* = 243).

Nonetheless, the findings show that most (46%) farmers indicated equal involvement for men and women in the extent to which their roles and responsibilities in maize production are clearly defined and respected. This is a positive step as it promotes equal access to food production resources, as such, facilitating the attainment of household food security. Furthermore, the findings show that farmers indicated more involvement of women than men in access to social protection programmes and services (44%) and in the address of gender-based violence and harassment (58%) in maize production settings. A related study (Das et al., 2023) argued that increased women’s involvement in food systems is linked to improved dietary diversity and nutrition outcomes for women themselves and their household members. This can be attributed to differences in spending habits of men and women, whereby women tend to spend much more on household food than men do.

### Gender integration perception levels during maize processing

Results show that the majority of farmers indicated less involvement of women than men when it comes to gender roles in maize processing being recognised and respected (71%); access to market information and trade opportunities in maize processing (70%); control over the processing and marketing of maize products (68%); access to credit and financing for maize processing activities (66%); opportunities to innovate and adopt new maize processing technologies (58%); and gender-specific needs^1^ and constraints being considered in maize processing facility design and equipment selection (56%). Furthermore, most of the farmers also indicated less involvement of women than men when it comes to gender contributions to maize processing being valued and compensated (48%); workloads and responsibilities in maize processing being distributed and recognised equally (39%); gender representation in maize processing roles, including management and leadership positions (38%); access to training and skills development in maize processing technologies (38%); and opportunities to participate in quality control and assurance processes (37%) (Figure 6). However, the FGDs reported that women are more responsible for maize processing activities like milling or making nsima (maize meal) flour than men are due to cultural norms.

**Figure 6:**
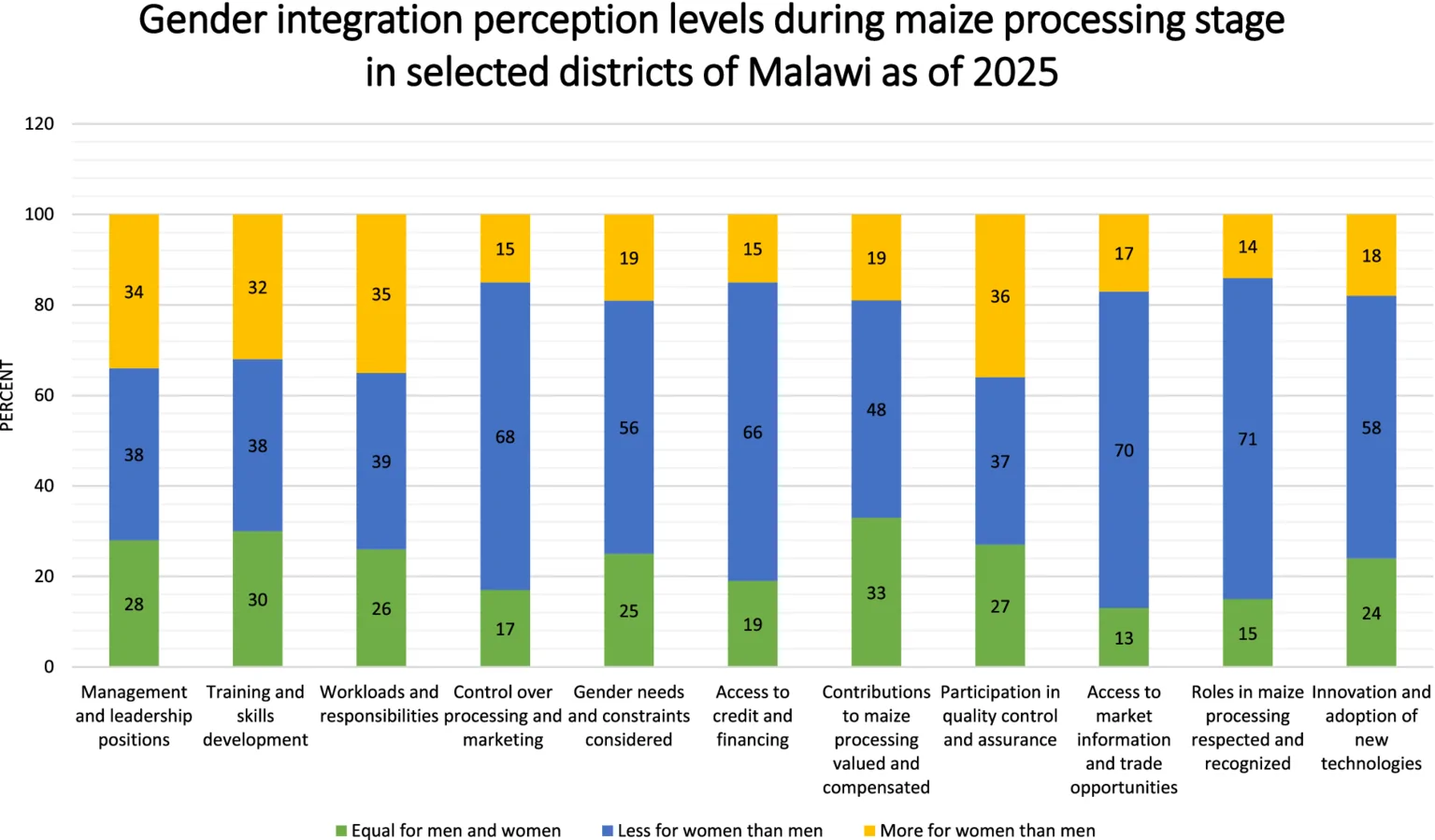
Gender integration perception levels during maize food processing (*n* = 243).

These findings show that women in Malawi participate less than men in maize processing activities. This has a negative implication, as it deprives them of an equal opportunity to participate and benefit from value additions of maize products like their counterparts, men. This is in agreement with findings from a similar study (Quisumbing et al., 2021) which reported that women in Malawi, Benin, Bangladesh and the Philippines participated less than men in agricultural value chains due to contextual and cultural factors. Low participation of women in value chains means low benefits on their side and a higher risk of food insecurity in their households. Another related study (Malapit et al., 2020) stated that women face challenges that deprive them of fair and equitable treatment and that their efforts in food systems, including in food processing, are minimally recognised. It is therefore important to engage in women empowerment initiatives that are tailored in promoting gender integration in all food system stages to enable women to fully benefit and attain food security.

### Gender integration perception levels during maize distribution

According to Figure 7, the majority of the farmers indicated less involvement for women than men when it came to access to transportation and logistics resources for maize distribution (73%); gender roles and responsibilities in maize distribution being clearly defined and respected (66%); control over the distribution channels and markets (65%); gender contributions to maize distribution being valued and compensated equally (63%); decision-making processes around maize distribution (62%); and gender perspectives considered in designing maize distribution systems (62%). Furthermore, most of the farmers also indicated less involvement for women than men when it came to access to technology and innovations in maize distribution (48%) and opportunities to benefit from maize distribution value chains (44%). This was corroborated with the FGDs which indicated that men are the main decision makers when it comes to selling maize and determining prices at the market.

**Figure 7:**
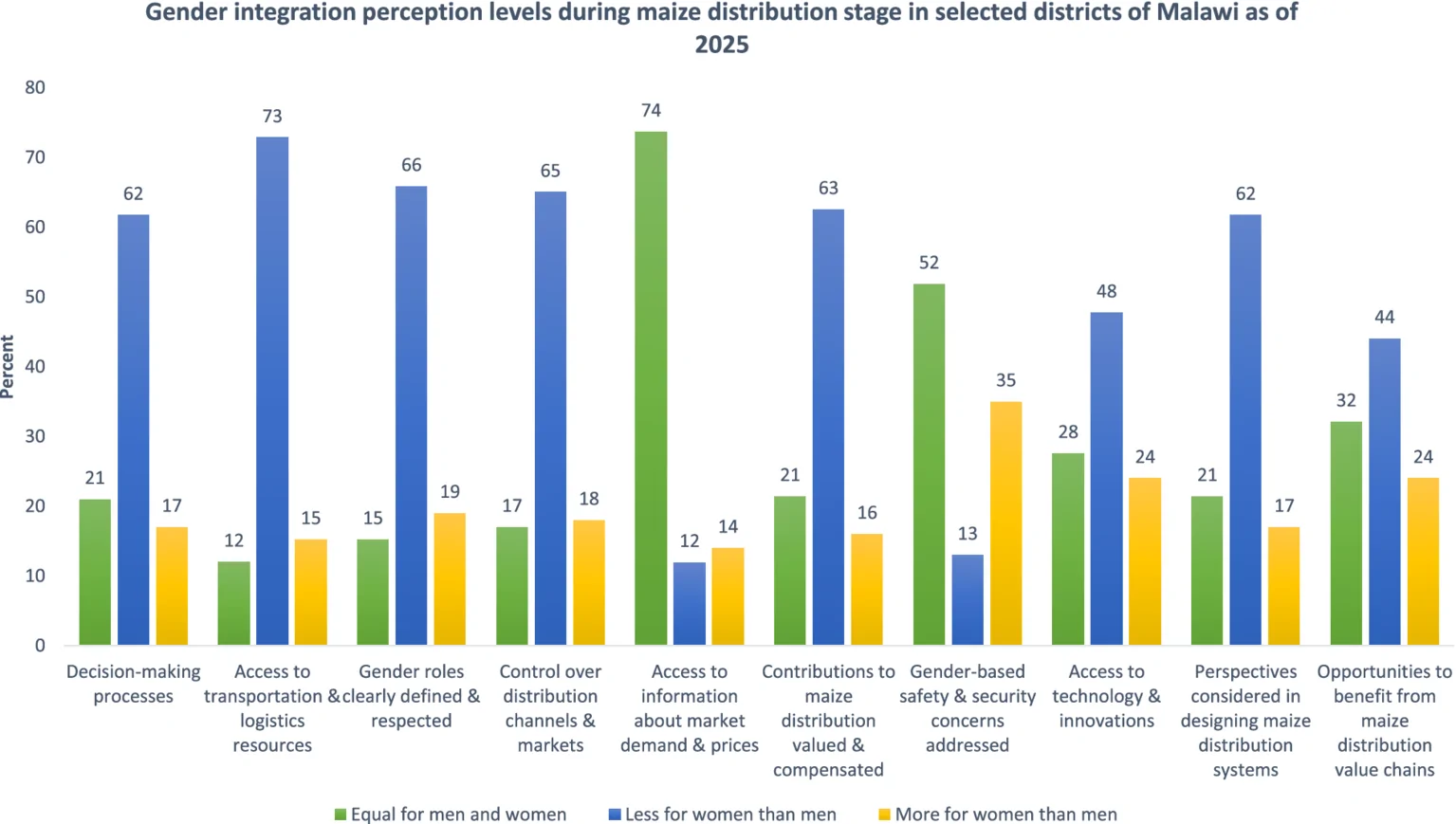
Gender integration perception levels during maize distribution (*n* = 243).

This means that women are less involved than men during the maize distribution stage of the maize food system. Less involvement of women during maize distribution can be associated with poor agricultural market systems that favour men over women in food distribution. A related study (Kihiu and Amuakwa-Mensah, 2021) argued that improved access to well-structured agricultural market systems for both males and females is more likely to improve food security of households and the country’s food basket at large. Another related study (Walther et al., 2019) revealed that poor capacity of women in fully taking part in the distribution of food crops predisposes them to poverty and food insecurity. To ensure adequate food security in communities, it is necessary that men and women be equally involved in food distribution. However, the majority of the farmers indicated equal involvement for men and women when it came to access to information about market demand and prices (74%) and gender-based safety and security concerns being addressed in food distribution workplaces (52%). This is a positive step toward gender integration in maize food systems, as improved access to well-structured agricultural market systems for both men and women is more likely to improve food security of households in communities (Kihiu and Amuakwa-Mensah, 2021).

### Gender integration perception levels during maize consumption

According to Figure 8, the majority of the farmers indicated more involvement of women than men in household food preparation and cooking responsibilities (89%); access to information about food safety and handling practices (78%); gender roles being recognised and valued (76%); gender rights to nutritious food and healthy eating being protected and promoted (72%); opportunities to benefit from food assistance programs and nutrition interventions (67%); opportunities to participate in food-related social and cultural activities (62%); consideration of food preferences and dietary needs in household food decisions (61%); consideration of gender perspectives in designing food storage and preservation technologies (60%); responsibility for food waste management and reduction (59%); and access to nutritious food and healthy eating options (58%). The FGDs corroborated this as they indicated that “women are responsible for preparing maize-based foods like nsima (maize meal) in the households. They are mostly assisted by female children in the homes”.

**Figure 8:**
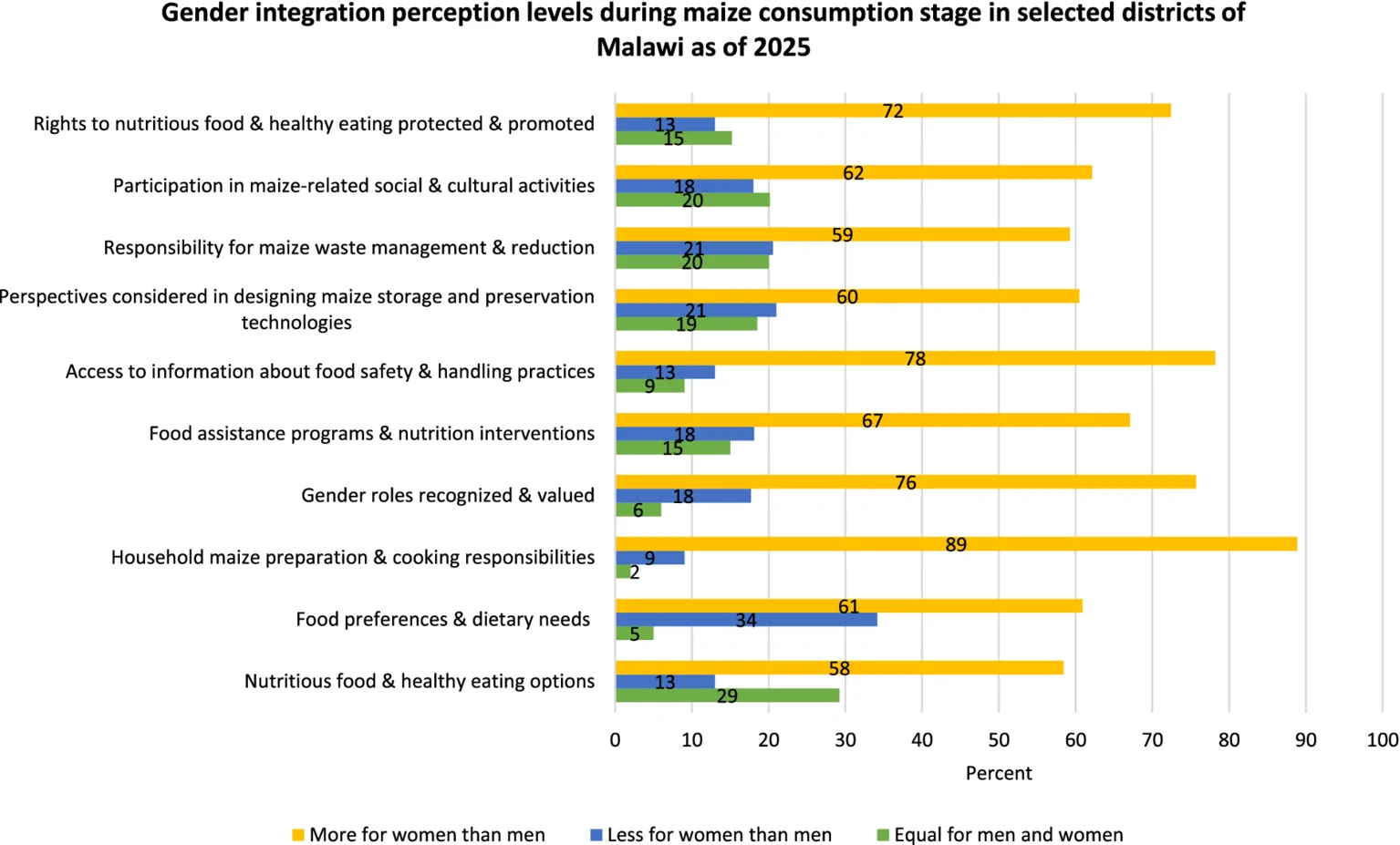
Gender integration perception levels during maize consumption (*n* = 243).

This means that women are more involved than men when it comes to household food consumption-related issues. This may be the case due to cultural factors that regard women as the key players in food preparation issues compared to men. Involvement of more women than men in household dietary issues has a risk of exposing the households to food insecurity. Previous studies (Argaw et al., 2021; Ashagidigbi et al., 2022) have linked sole dietary decision-making by women to increased probability of poor diets due to their low incomes and food production capacities. Promoting equal participation of men and women in household dietary issues can enhance food and nutrition security. Previous studies (Gebre et al., 2021; Mohammed et al., 2023) have argued that joint household decision-making is critical for improved household food security outcomes. Malawi should promote joint decision-making between men and women on household dietary issues as a way of promoting household food and nutrition security. A similar study (Visser and Wangu, 2021) pointed out that women are pivotal to food systems, both as primary food producers and as primary caretakers of households, and that as such it is important to promote gender integration.

### Correlations between maize food system stages variables

Results on correlation between maize food system stages’ variables are presented in Table 3. There was a weak positive correlation between levels of gender integration in decision-making processes throughout the maize production stage and gender integration levels in maize processing roles, including management and leadership positions. This means that equal involvement of women and men in decision-making at the maize production stage does not strongly predict equal involvement of women and men in maize processing roles, as the two maize system stages appear to operate somewhat independently. Furthermore, there was a weak positive correlation between gender integration levels in maize processing roles, including management and leadership positions, and the extent to which women and men were equally involved in decision-making processes around maize distribution. This means that whether women and men were equally represented in maize processing roles had very little positive connection to whether they were equally involved in decision-making around maize distribution. This strengthens the need for stage-specific gender strategies to improve gender integration. Lastly, there was a weak but significant positive correlation between the extent to which women and men were equally involved in decision-making processes around maize distribution and the extent to which women and men shared household maize preparation and cooking responsibilities equitably. This means that when women and men were more equally involved in deciding about maize distribution, there was also a slight tendency for women and men to share household cooking more equally. A previous study (Perelli et al., 2024) argued that gender integration at agricultural technology adoption stage, ensures continued involvement of joint men and women in the subsequent stages of food systems. This agrees with a related study (Gebre et al., 2022) which claimed that joint decision-makers received higher prices on each stage than sole female or male decision-making farmers as they had stronger bargaining power in the market. Deliberate policies should be formulated to promote equal participation of women and men in all maize food system stages to ensure equal participation and benefits between men and women.

**Table 3:** Correlations between maize food system stages’ variables (*n* = 243).

Variable	Statistic	Decision-making in maize production chain	Representation in maize processing roles	Decision-making around maize distribution	Household maize preparation and cooking responsibilities
Decision-making in maize production chain	Pearson correlation	1	0.259^**^	0.321^**^	0.182^**^
	Sig. (2-tailed)		0.000	0.000	0.005
Representation in maize processing roles	Pearson correlation	0.259^**^	1	0.250^**^	0.321^**^
	Sig. (2-tailed)	0.000		0.000	0.000
Decision-making around maize distribution	Pearson correlation	0.321^**^	0.250^**^	1	0.249^**^
	Sig. (2-tailed)	0.000	0.000		0.000
Household maize preparation and cooking responsibilities	Pearson correlation	0.182^**^	0.321^**^	0.249^**^	1
	Sig. (2-tailed)	0.005	0.000	0.000	

**Correlation is significant at the 0.01 level (2-tailed).

## Conclusions and recommendation

Results of this study have shown that the majority of members in farmer groups and cooperatives, loan beneficiaries, and farm inputs beneficiaries are males. This shows that males are the dominant sex in the gender composition of farmer groups and cooperatives and that they have an upper hand in accessing resources critical for maize production compared to women. Men take major roles in deciding on how to access farm inputs and how to use them at household level. Furthermore, men and women jointly take major roles in deciding on the type of seed to grow and on the use of loan money. This shows that men take a centre stage in the decision-making processes of their households. Women are less involved than men when it comes to activities during the maize production, processing, and distribution stages. However, there is higher involvement of women than men when it comes to activities under the maize consumption stage. Correlation tests have shown weak linkages and little influence between the food system stages. The study interviewed household heads only, the majority of whom were male. Household headship in the Malawian context is frequently likened to male dominion as such the findings may over-represent male views. Furthermore, a cross-sectional approach was used, it makes it incapable of tracking changes over time. The study also focussed on the central and northern regions and excluded the southern region, which may affect generalisability of the findings. Future studies should focus on involving multiple regions and longitudinal methods to enhance the depth and breadth of the research, generalisability, and accuracy of the findings. Future studies should also investigate gender roles in other staple crops and assess policy impact on gender integration in food systems. Lastly, policymakers should make deliberate policies to promote equal access to training, credit, cooperative support, participation and benefits between men and women in maize food systems.

## Data Availability

Data supporting the conclusions made in this manuscript has been uploaded online through Zenodo.org and has been cited as follows: Munkhuwa, V. (2026). Gender roles in maize food systems dataset for Victor Munkhuwa [Dataset]. Zenodo. http://doi.org/10.5281/zenodo.22529660 (Munkhuwa, 2026).

## Footnotes

1 Access, usability, time, and control.
